# Nanoparticle Delivery and Tumor Vascular Normalization: The Chicken or The Egg?

**DOI:** 10.3389/fonc.2019.01227

**Published:** 2019-11-12

**Authors:** George Mattheolabakis, Constantinos M. Mikelis

**Affiliations:** ^1^School of Basic Pharmaceutical and Toxicological Sciences, College of Pharmacy, University of Louisiana Monroe, Monroe, LA, United States; ^2^Department of Pharmaceutical Sciences, Texas Tech University Health Sciences Center, School of Pharmacy, Amarillo, TX, United States

**Keywords:** nanoparticles, delivery, tumor, vessel, normalization

## Abstract

Tumor-induced angiogenesis has been a significant focus of anti-cancer therapies for several decades. The immature and “leaky” tumor vasculature leads to significant cancer cell intravasation, increasing the metastatic potential, while the disoriented and hypo-perfused tumor vessels hamper the anti-tumor efficacy of immune cells and prevent the efficient diffusion of chemotherapeutic drugs. Therefore, tumor vascular normalization has emerged as a new treatment goal, aiming to provide a mature tumor vasculature, with higher perfusion, decreased cancer cell extravasation, and higher efficacy for anti-cancer therapies. Here we propose an overview of the nanodelivery approaches that target tumor vasculature, aiming to achieve vascular normalization. At the same time, abnormal vascular architecture and leaky tumor vessels have been the cornerstone for nanodelivery approaches through the enhanced permeability and retention (EPR) effect. Vascular normalization presents new opportunities and requirements for efficient nanoparticle delivery against the tumor cells and overall improved anti-cancer therapies.

## Introduction

**Figure 1:**
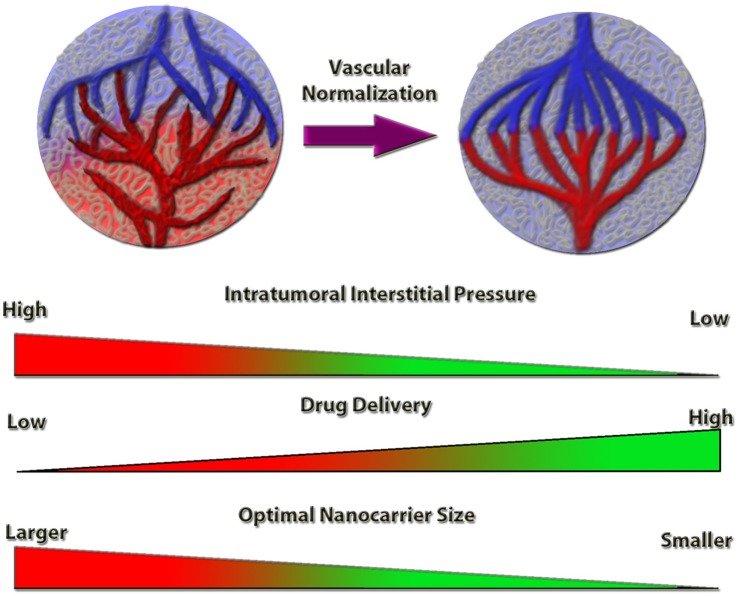
Schematic representation of the tumor vessel normalization's impact on intratumoral interstitial pressure and drug delivery efficiency. Normalized tumor vessels decrease tumor hypoxia and intratumoral interstitial pressure, which increases the anti-cancer drug delivery efficiency. Nanocarrier size is a limiting factor for optimal targeting and delivery, the efficiency of which is inversely proportional to the nanocarrier size, under vessel normalization conditions.

Anti-angiogenic therapy has been a major focus area of anti-cancer research for several decades (1). Blocking the immature, disorganized tumor-derived vessels led to significant tumor inhibitory effects in preclinical models and rendered anti-angiogenic therapy as a promising approach for cancer treatment, especially in combination with chemotherapy. A large volume of preclinical data with angiogenesis inhibitors led to the FDA approval and release of anti-angiogenic therapies in the clinic (2, 3). The most characteristic target is vascular endothelial growth factor (VEGF), where anti-VEGF therapy, such as bevacizumab, a humanized monoclonal anti-VEGF antibody, or sorafenib and sunitinib, VEGF receptor tyrosine kinase inhibitors, were incorporated in anti-cancer treatment options either as single agents or adjuvant therapy (3, 4). However, the clinical outcome of anti-angiogenic therapy did not meet the expectations: although progression-free survival was increased in some cases, such as metastatic colorectal (5) and ovarian cancer (6), or renal cell (7) and hepatocellular carcinoma (8), in other cancers, such as breast, melanoma, pancreatic and prostate, progression-free survival and overall survival were not increased (4, 9). The main pitfall of anti-angiogenic treatment is the impaired tumor perfusion, which limits the access to chemotherapeutic agents, impedes the tumoricidal activity of immune cells, and increases hypoxia, further driving tumor aggressiveness and metastasis (Figure 1) (4, 10).

The rapid growth of solid tumors induces the secretion of angiogenic factors by the tumor cells to accommodate the needs of their increased proliferation rate. This results to the rapid development of imperfect vascularization in the tumor area, characterized by tortuous and leaky vessels. The imperfections of the rapidly growing vasculature have been identified as porous-like structures of ~400–600 nm in diameter, leading to the enhanced permeability and retention (EPR) effect (11). The features of the EPR include a size-dependent accumulation of molecules and structures due to the leaky vessels, where particles (such as nanoparticles) and macromolecules will diffuse out of the tumor vessels that bear the imperfections, compared to any healthy tissue, where these imperfections are absent. This can induce an augmented drug concentration in the tumor area, further supported by the impaired lymphatic drainage associated with the abnormal tumor vasculature (12).

The limited clinical outcome of anti-angiogenic therapy has driven the last decade the concept of vascular normalization as a complementary therapeutic approach for anti-cancer treatment. Normalization of tumor vasculature is expected to provide a properly oriented, well-constructed vascular network with reduced vascular density, increased perfusion and limited hypoxia, which will lead to better drug delivery and anti-cancer efficacy (13, 14). An increasing number of studies demonstrate the promising outcome of vascular normalization strategies. Lower doses of current anti-angiogenic therapies, such as bevacizumab, are reported to achieve tumor vessel normalization (4, 13). Even in aggressive tumors, such as glioblastoma, treatment with cediranib, an anti-angiogenic agent, improved vessel perfusion in a subset of patients and increased overall survival (15). Tumor vessel normalization has also been achieved by non-pharmacological approaches; aerobic exercise can drive vascular normalizing effects and improve chemotherapeutic efficacy. The leading player, in that case, is considered to be the shear stress, which, when increased, enhances vascular integrity through secretion of vascular normalization mediators, such as thrombospondin-1 (16–19).

The limitations of vascular normalization approaches follow, to a certain extent, the limitations of anti-angiogenic therapy. The most common is the evasive resistance, the acquired resistance of the endothelium towards anti-angiogenic therapy that targets a growth factor, by upregulating others, which will compensate for its inhibition (4). The main goal for tumor vascular normalization is the improvement of anti-cancer drug delivery. However, the window for anti-cancer therapy is normally short, not easily identifiable, and does not occur uniformly in the patient population (13, 14). The extension and identification of this therapeutic window consist the primary goal of current studies focusing on vascular normalization, and one of the main goals is the identification of markers denoting the potent therapeutic window for vascular normalization. An example is Anterior gradient 2, a plasma protein secreted from tumor cells, which was proposed as a vascular normalization marker during anti-angiogenic treatment (20). Certain approaches to overcome that therapeutic window have been proposed, such as the simultaneous administration of anti-angiogenic and chemotherapeutic drugs through nanoparticle delivery (21).

Nanodelivery methods ensure the selective targeting of a specific tissue, offering improved delivery with significantly fewer side-effects (11). Nanoformulations are known to significantly enhance the effect of certain compounds compared to direct administration (22) and nanodelivery methods are incorporated into therapeutics of multiple diseases, including cancer (23). In this mini-review we summarize the current knowledge from studies, where nanodelivery is utilized for or in conjunction with tumor vascular normalization and discuss advantages, limitations and potentials.

## Nanoformulations and Cancer Nanotherapeutics

Traditional drug delivery systems have been unable to address the complex therapeutic and physicochemical necessities presented by the traditional and new active molecules, including poor aqueous solubility, poor specificity, unfavorable pharmacokinetics and high toxicity (11). Nanotechnology has steadily grown to a promising field of research and application for the diagnosis and treatment of various diseases, among which is cancer. Nanocarriers are colloidal systems used for drug delivery, capable of entrapping, encapsulating and delivering active molecules to tissues and cells (11). As their name suggests, nanocarriers have a particle size at the submicron range (<1 μm), though it is generally regarded that nanocarriers used through systemic administration will typically have a size below 200–250 nm. This stems from the natural filtering mechanism of the body, where nanoparticles of larger dimensions are retained and removed from the circulation through splenic filtration (24). Nanotechnology has yielded significant advantages over traditional pharmaceutical formulations, such as: (a) improved drug stability; (b) improved pharmacokinetics/biodistribution; (c) reduced non-specific toxicity; (d) reduction in drug dosage and dosing frequency, and; (e) high drug loading for compounds insoluble in water (11).

The growing field of nanotechnology has yielded new and innovative carriers with distinct and multifaceted properties, while new formulations and approaches are constantly being developed. We provide here a brief overview of the most important aspects of nanotechnology, describing the most frequently studied nanocarriers. Though there are overlaps or combinations of the technological advancements, the classification of the nanocarriers typically relies on their composition, having three major categories: (a) lipid-based; (b) polymer-based, and (c) inorganic nanocarriers (25).

Among the lipid-based formulations, liposomes are the best known and studied nanocarriers. They have achieved broad recognition for their capacity to protect and deliver active compounds, with improved biodistribution profiles and reduced toxicity (11, 25). Liposomes are primarily used for hydrophilic compounds, though their lipid bilayer allows the entrapment of hydrophobic compounds as well. More importantly, liposomal formulations have received FDA approval for use in cancer treatment, i.e., liposomal formulation of doxorubicin—Doxil^®^ (26), among others, which constitutes them as a reliable, safe, tested, and thus attractive nanocarrier model for human treatment or new drug development (27, 28).

Solid lipid nanoparticles (SLNs) are lipid-based nanocarriers, commonly prepared by dispersing melted solid lipids in water in the presence of a stabilizing emulsifier (29). Similarly to the oil-in-water (o/w) emulsions, the hydrophobic environment inside the nanocarriers makes them ideal for the entrapment and delivery of molecules with low aqueous solubility, though, in contrast to the o/w emulsions, the hydrophobic core is solid and not liquid (29). Unlike o/w emulsions, which require oils that may present significant toxicity or biocompatibility limitations (30), both the liposomes and the SLNs utilize lipids commonly found in cells (i.e., phospholipids) that can be of natural source or synthetically made/modified. Not surprisingly, liposomes and SLNs are considered biocompatible and biodegradable, with an excellent safety record (31). In fact, synthetic approaches using polymer synthesis and chemical attachment of antibodies or targeting moieties have advanced the development of multifunctional lipids for long-circulating nanocarriers that may actively target a variety of cells, such as macrophages, endothelial or tumor cells (32–34).

Similarly, polymer-based nanocarriers have emerged as promising nanocarriers for drug delivery. The progress on polymer chemistry has allowed the development of new polymer structures with multi-faceted and highly adjustable properties, advancing the development of nanosized micelles, solid-core nanoparticles, polymersomes and dendrimers (11). These carriers have tunable characteristics, defined by the physicochemical properties of the used polymer or combination of polymers, capable of delivering unstable hydrophilic and hydrophobic compounds, or molecules that otherwise would not be capable of crossing the cell membrane, such as nucleic acids (i.e., si/miRNAs and plasmids).

Finally, inorganic nanoparticles are frequently composed of magnetic iron oxide, silica oxide and gold, among other materials. Similar to the other categories, the inorganic nanoparticles can be surface-modified to achieve long-circulating properties, actively target specific cells and tissues, and protect active compounds. Furthermore, inorganic nanoparticles, such as iron oxide/magnetic nanoparticles, can respond to external stimuli, such as magnetism, which permits their detection or active targeting to specific parts of the body, or demonstrate unique optical properties for improved *in vivo* imaging, such as quantum dots and up-converting nanoparticles, which lipids and polymers cannot provide (35–37).

The efficacy of nanodelivery in different tumors largely varies, guided by the variable tumor vascular characteristics, such as vessel architecture, interstitial fluid and extracellular matrix composition, phagocyte infiltration and presence of necrotic areas. Parameters, such as the extravasation of the nanoparticles from tumor blood vessels, their diffusion through the extracellular tissue and their interaction with the tumor microenvironment constitute the EPR effect, elegantly analyzed by Bertrand et al. (23). The EPR effect in solid tumors was initially described ~3 decades ago, and was one of the driving forces for the scientific advancements taking place in the field of nanotechnology. The goal of nanotechnology-based treatment is to utilize or enhance the EPR effect in tumors, allowing better pharmacological targeting of the tumor tissue, leading to an increasing build-up of the nanocarriers with the active compound to the tumor area, which is further supported by the impaired lymphatic drainage in solid tumors (38). Alternatively, sonoporation, the combination of ultrasound and microbubbles, has improved liposome accumulation and their penetration through the tumor vasculature into the tumor interstitium (39).

The EPR effect has received criticism recently, regarding its significance in the passive targeting to tumors, its dependency on the stage and the type of tumor (40), and whether it is present in human tumors (41). There is a potential sift on the paradigm on the use of nanoformulations and their drug delivery capacity under rapidly growing vs. slowly growing tumors, as well as the influence of the vascular architectural structure. Below we summarize the up-to-date literature for nanotherapeutics targeting vessel normalization and their potential for anti-angiogenic therapies.

## Vessel Normalization

The need for vascular normalization has been further highlighted with the recent advances in tumor immunotherapy. Several antibodies targeting the immune checkpoint proteins, such as pembrolizumab, nivolumab and ipilimumab have been approved for clinical practice (42–44), and immune checkpoint inhibitions consist a revolutionary anti-cancer approach for solid tumors (45). However, a subset of patients does not benefit, and the reasons are not known (46). A potential reason for the ineffectiveness of tumor immunotherapy for the non-responding patients could be the inability of the immune cells to sufficiently access the tumor mass, and tumor vascular normalization looks a promising solution (14, 47).

A groundbreaking study for nanodelivery and tumor vasculature normalization was from Rakesh Jain's lab, where they showed that vascular endothelial growth factor receptor-2 (VEGFR2) targeting led to tumor vessel normalization and the subsequent decrease of the intratumoral interstitial pressure, improving nanoparticle delivery. It was also demonstrated that smaller nanoparticles, of 12 nm diameter, are more potent to invade rapidly to the tumor area than the larger ones (48). Although the increasing optimization of surface modifications renders these size constrains not easily applicable in biomedical applications (49), it was later demonstrated that tumor vascular normalization through VEGFR2 inhibition improved accumulation of also larger nanoparticles, of 20 and 40 nm size, in the tumoral bed. However, inside the tumor, smaller nanoparticles presented a more homogeneous distribution (50).

Increased tumor vascularity increases nanoparticle delivery, but increased collagen deposition, which also leads to increased interstitial pressure, is an inhibitory factor (51). For this, recent attempts to induce tumor vessel normalization targeted both the tumor microenvironment, as well as the extracellular matrix (ECM). An example is the co-administration of antibodies targeting vascular endothelial growth factor (VEGF) and transforming growth factor β1 (TGF-β1), which led to a combined vascular and ECM normalization and thus improved intratumoral nanomedicine delivery (52).

Gold nanoparticles have been studied for vascular normalization in several tumor types. Endostatin is an endogenous angiogenesis inhibitor. Gold nanoparticle-encapsulated human recombinant endostatin led to a transient tumor vascular normalization in non-small cell lung cancer. Chemotherapy administered during the normalization window was significantly more potent than when administered as a monotherapy (53). Gold nanoparticles have been successfully used to block metastasis in melanoma by increasing tumor vascular normalization (54). Treatment of cediranib, a vascular endothelial growth factor receptor inhibitor, normalized tumor vessels in a breast cancer model, enhancing tumor retention of enzyme responsive size-changeable gold nanoparticles, further demonstrating that combinatorial treatment could be a potent approach for efficient tumor diagnosis and treatment (55).

Epidermal growth factor receptor (EGFR) tyrosine kinase inhibitors, such as erlotinib, are considered effective therapies for EGFR mutation positive non-small cell lung cancers. The promising outcome is often compromised by resistance driven by upregulation of the anti-apoptotic protein survivin, in the cancer cells. A novel approach using chloroquine to normalize the tumor vasculature, combined with anti-EGFR aptamer-mediated delivery of erlotinib and survivin shRNA co-administration significantly hampered tumor growth (56).

Nogo-B is a potent growth factor mediating endothelial cell functions, such as wound healing angiogenesis and chemotaxis, through binding to the Nogo-B receptor (57). Nogo-B receptor knockdown was achieved through nanoparticles with charge convention in the acidic tumor microenvironment, leading to breast cancer vessel normalization *in vivo* and inhibition of metastatic incidence (58).

Cyclooxygenase-2 (COX-2) is upregulated in several cancer-related pathways regulating cell proliferation, apoptosis, multi-drug resistance and angiogenesis (59). Celecoxib, a clinically-relevant COX-2 inhibitor, was reported to normalize the tumor microenvironment, including the tumor vessels, thus improving the uptake of paclitaxel-loaded micelles in xenografts of human lung adenocarcinomas (60).

Brain vascular normalization and blood-brain barrier restoration are important for glioblastoma. Liposomal formulation of the chemotherapeutic drugs irinotecan, doxorubicin and vincristine improved their pharmacokinetic profile and increased their potency in tumor inhibition. Apart from the size, mostly irinotecan- treated tumors led to vascular normalization, characterized by increased perfusion, assessed by Hoechst uptake, decreased extend of the discontinuous basement membrane, increased number of pericyte-covered capillaries and decreased vessel diameter (61).

miRNAs play a major role in tumor aggressiveness and metastasis. miRNA-200 was initially reported to block epithelial-mesenchymal transition (EMT) in tumors through *ZEB1* and *ZEB2* downregulation (62–64). miRNA-200 blocks tumor angiogenesis through IL-8 and CXCL1 inhibition. Nanoparticle-mediated miRNA-200 delivery reduced tumor angiogenesis and induced tumor vessel normalization, leading to tumor growth and metastasis inhibition in ovarian, lung, renal and breast adenocarcinomas (65).

Nanoparticle-based approaches are used not only for the delivery of vascular normalizing agents, but also for their development and evaluation. An example is NGR-TNF, a chimeric protein that couples the tumor homing peptide CNGRCG, which targets aminopeptidase N or myeloid plasma membrane glycoprotein CD13, also expressed in angiogenic vessels, with the N-terminus of the tumor necrosis factor-α (TNF). It is a vascular targeting agent, which presents antitumor effects and is in clinical trials for tumors either as monotherapy or in combination with chemotherapeutic drugs. Low dose treatment inhibited angiogenesis by inducing endothelial cell apoptosis, whereas at the later stages it led to tumor vascular normalization, assessed by the increased pericyte and smooth muscle cell coverage. The CD31 targeting was verified *in vivo* by coupling of the CNGRCG peptide to fluorescent nanoparticles (quantum dots, described above) (66). The studies are summarized in Table 1.

**Table 1:** Table summarizing the data regarding vascular normalization, including tumor models, molecular targets, targeting agents, and nanoformulations.

**Tumor model(s)**	**Molecular target**	**Targeting agent**	**Nanoformulation**	**References**
4T1, E0771 (breast cancer)	VEGFR2	DC101 Ab	Quantum Dots^*^	(48)
MCaP0008 (breast adenocarcinoma)	VEGFR2	DC101 Ab	Quantum Dots-mPEG^*^	(50)
GL261 (glioblastoma)	VEGFR2, TGF-β1	DC101, anti-TGFβ1 Abs	Quantum Dots-mPEG^*^	(52)
H22 (hepatocellular carcinoma)	VEGFR2, integrins, nucleolin	Endostatin	Gold nanoparticles-PEG (AuNPs-PEG)	(53)
B16-F10 (melanoma)	–	–	Gold nanoparticles-(AuNPs)	(54)
4T1 (breast cancer)	VEGFR2	cediranib	Enzyme responsive-size-changeable gold nanoparticles (AuNPs-A&C)	(55)
H1975 (non-small cell lung cancer)	EGFR, survivin	Erlotinib, survivin-shRNA	PAMAM dendrimers with anti-EGFR aptamers	(56)
4T1 (breast cancer)	Nogo-B receptor (NgBR)	NgBR siRNA	PLGA-PEI-DMMA nanoparticles	(58)
A549 (lung cancer)	Cyclooxygenase-2 (COX-2)	Celecoxib	Paclitaxel-loaded Micelles^*^	(60)
U251MG (glioblastoma)	Topoisomerase 1, microtubules, topoisomerase 2	Irinotecan, vincristine, doxorubicin	liposomes	(61)
344SQ (lung cancer), HeyA8, A2774 (ovarian cancer), BT549 (breast cancer)	CXCL-1, IL-8	miRNA-200	DOPC and RGD-CH-NP nanoparticles	(65)
RIP-Tag2 (pancreatic cancer)	TNFR1 and 2	NGR-TNF (TNF-α with CD13-targeting peptide)	Quantum Dots^*^	(66)

* *Study where nanoparticles were used, not for the transfer of the targeting agent for vascular normalization, but for anti-cancer or imaging purposes*.

## Discussion

It is important to note that tumor vessel normalization does not automatically correspond to better distribution of all nanodelivery systems. Tumor vessel normalization induced by imatinib mesylate limited the distribution of large (~110 nm) but enhanced the distribution of small (~23 nm) nanoparticles in human lung adenocarcinoma. However, the nanoparticle distribution inside the tumors was overall reduced, compared to that of micelles, and micelle-based delivery of paclitaxel significantly improved its potency (67).

For the concept that vessel normalization is significantly affecting the efficiency for drug delivery using nanoformulations, nanotechnology has undoubtedly allowed the delivery, protection and targeting of compounds that other drug formulations (i.e., implants, microparticles, free drug) are incapable of achieving (68). The controversial EPR effect, along with the vessel normalization approaches, only illustrate the potential of new methodologies, such as smaller nanocarriers and active targeting. It is now widely accepted that mild anti-angiogenic therapy leads to tumor vessel normalization and tumor vessel normalization induces the uptake of nanoparticle-based delivery, leading to more potent anti-tumor activity (48, 69). This process is also accompanied by mathematical models simulating the events and predicting the penetrance of drugs into the tumor area (70). There is significant potential for novel compounds treating the vascular endothelium to be actively delivered by nanocarriers to the tumor area, safely, with reduced toxicity and high specificity, while avoiding *in vivo* degradation (68). It is the authors' opinion that nanotechnology will play a significant role in the development of these therapies in the future. With the existence of several biological barriers for the successful delivery of active molecules and nanocarriers, some of which we described here, the optimal physicochemical parameters of the nanocarriers will need to be carefully considered, with their size and shape being paramount (48, 50). Finally, the combination of surface modification for cellular specificity and the achieved vascular normalization may enhance and prolong nanocarrier presence in the tumor microenvironment for improved pharmacological activity. Overall, nanoparticle-mediated drug delivery targeting both tumor cells and tumor vessels could be a promising approach for efficient anti-cancer therapies.

## Author Contributions

GM and CM contributed to the conception of the article, wrote and revised the final manuscript, and agreed on its submission to this journal.

### Conflict of Interest

The authors declare that the research was conducted in the absence of any commercial or financial relationships that could be construed as a potential conflict of interest.
